# Portrayal of a young woman in 16^th^ century Islamic art: Does she have anti-N-methyl-D-aspartate receptor (anti-NMDAR) encephalitis?

**Published:** 2018-10-07

**Authors:** Daniel Kondziella, Sara Bech

**Affiliations:** 1 Department of Neurology, Rigshospitalet Hospital, Copenhagen University, Copenhagen, Denmark; 2 Department of Clinical Medicine, University of Copenhagen, Denmark; 3 Department of Neuroscience, Norwegian University of Science and Technology, Trondheim, Norway; 4 Department of Neurology, Bispebjerg Hospital, Copenhagen University, Copenhagen, Denmark

**Keywords:** Autoimmune Encephalitis, Anti-N-Methyl-D-Aspartate Receptor Encephalitis, History of Medicine, Neurology, Art, Psychosis

Anti-N-methyl-D-aspartate receptor (Anti-NMDAR) encephalitis was first reported in 2007.^1^ Soon, it was realized that this entity was more frequent in young adults than infectious encephalitides, and that it could explain most of previously cryptogenic cases of non-infections encephalitis in the intensive care setting.^2^^,^^3^ It follows that many cases of anti-NMDAR encephalitis must have been missed in the past. However, the origins of anti-NMDAR encephalitis (i.e., where and when it occurred for the first time) are entirely unknown. Here, we present a historic case with several core features of anti-NMDAR encephalitis, suggesting that this disorder might have existed for more than 500 years. 

Zulaykha, a young woman in her teens or twenties, develops subacute onset of neuropsychiatric features (delusions, hallucinations, and emotional dissociative behavior) together with neurological symptoms, including extrapyramidal signs (posturing, motor agitation, and disinhibited behavior), followed by spontaneous remission after 12 months, yet resulting in severe long-term deficits with cognitive and mood disturbances. We argue that this presentation is compatible with a disease course as typically seen with anti-NMDAR encephalitis.

Figure 1 reveals a miniature, entitled “Zulaykha is mad with longing after having seen Yusuf in a dream for the second time”. The painting is attributed to Qazvin (Iran, 1581) and illustrates Jami’s famous poem “Yusuf and Zulaykha” from 1483.^4^

The story of Yusuf and Zulaykha is known throughout world literature;^4^ Zulaykha, a beautiful young princess in her late teens or early twenties, has recurrent dream visions about handsome Yusuf. She is overwhelmed with such mad love that she becomes psychotic, and attempts to flee from the court. 

**Figure 1:**
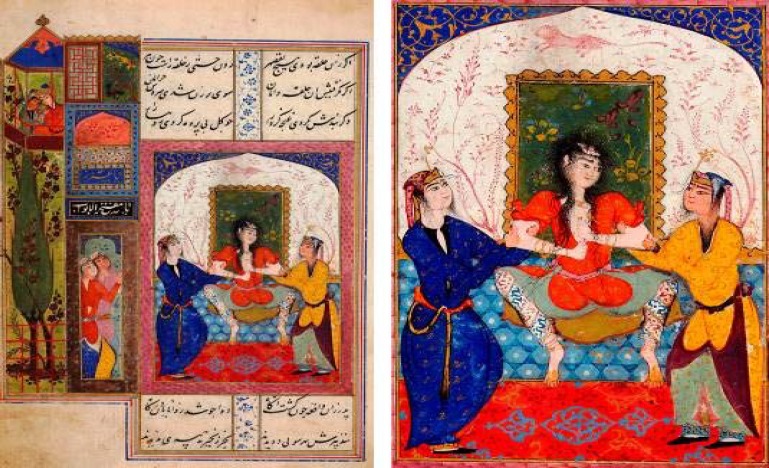
This miniature, entitled “Zulaykha is mad with longing after having seen Yusuf in a dream for the second time”, is attributed to Qazvin (Iran, 1581) and illustrates Jami’s famous poem “Yusuf and Zulaykha” from 1483. In the left upper corner, an intimate scene of a loving couple symbolizes the union with her beloved Yusuf which the princess longs for so much (Left panel). The magnification shows Zulaykha in despair and being restraint physically by two of her servants (Right panel). The painting is on display at the David Collection, Copenhagen, Denmark (106b/2006; reprinted with permission; courtesy of Pernille Klemp).

However, the king has his daughter shackled with a golden chain (a glimpse of which can be seen over Zulaykha’s henna-stained feet), and she is kept in this state for an entire year. Although Zulaykha slowly recovers, her life is ruined by unreturned love, and she ends up old and alone. 

As an art historian notes, the painting is remarkable for its “realistic illustration of madness”.^4^ Zulaykha displays “unconventional stance with legs apart”; “her hair falls unkempt in long curls”; and “her long underwear is revealed”.^4^ She “attempts to tear her clothes apart and bare her breast”, but is stopped by her servants. Of note, while her motor agitation is clearly depicted, Zulaykha’s face with its “lack of expression” is strikingly blank, suggesting “a state of mental dissociation”.^4^

For obvious reasons, we cannot exclude the possibility that Zulaykha suffered from schizophrenia or another non-inflammatory psychotic disorder. However, major clinical features of anti-NMDAR encephalitis can be identified in the painting, and the story behind it. The age and sex of the protagonist (a young woman), and the subacute onset of neuropsychiatric symptoms, including repeated episodes of hallucinations and disinhibited behavior, are compatible with anti-NMDAR encephalitis.^1^^-^^3^ In addition, pronounced motor agitation, necessitating physical restraint, yet with dissociative features illustrated by a blank facial expression, and extrapyramidal signs, including inappropriate and bizarre stances compatible with posturing, are characteristic for this disorder.^1^^-^^3^ Finally, the prolonged time course over many months followed by spontaneous remission, yet resulting in severe chronic cognitive and behavioral long-term consequences with impaired social functioning, suggests that Zulaykha may have suffered from anti-NMDAR encephalitis.^1^^-^^3^

Artists and poets have occasionally depicted brain conditions long before these disorders came to the attention of psychiatrists and neurologists.^5^ We will never know if our diagnosis of anti-NMDAR encephalitis is correct but, of note, the painting is consistent with the idea that anti-NMDAR encephalitis may have existed for many centuries, irrespective of cultural or geographic boundaries.

## Notes:

**How to cite this article:** Kondziella D, Bech S. Portrayal of a young woman in 16^th^ century Islamic art: Does she have anti-N-methyl-D-aspartate receptor (anti-NMDAR) encephalitis? Iran J Neurol 2018; 17(4): 192-4.
